# Supplementary material for: Cubic 3D Chern photonic insulators with orientable large Chern vectors
Source: Nat Commun. 2021 Dec 17;12:7330. doi: 10.1038/s41467-021-27168-w (PMC8683445; doi:10.1038/s41467-021-27168-w)
Supplement: Supplementary file 1 — Supplementary Information [file 41467_2021_27168_MOESM1_ESM.pdf]

Supplementary Information for 'Cubic 3D Chern photonic insulators with orientable large Chern vectors'

Chiara Devescovi,[1,*] Mikel García-Díez,[2,1] Iñigo Robredo,[3,1] María Blanco de Paz,[1] Jon Lasa-Alonso,[4,5] Barry Bradlyn,[6] Juan L. Mañes,[7] Maia G. Vergniory,[1,8,9,†] and Aitzol García-Etxarri[1,8,‡]

[1]*Donostia International Physics Center, 20018 Donostia-San Sebastián, Spain*
[2]*Physics Department, University of the Basque Country (UPV-EHU), Bilbao, Spain*
[3]*University of the Basque Country (UPV-EHU), Bilbao, Spain*
[4]*Centro de Física de Materiales, Paseo Manuel de Lardizabal 5, 20018 Donostia-San Sebastian, Spain*
[5]*Donostia International Physics Center, Paseo Manuel de Lardizabal 4, 20018 Donostia-San Sebastian, Spain*
[6]*Department of Physics and Institute for Condensed Matter Theory, University of Illinois at Urbana-Champaign, Urbana, IL, 61801-3080, USA*
[7]*Physics Department, University of the Basque Country (UPV/EHU), Bilbao, Spain*
[8]*IKERBASQUE, Basque Foundation for Science, Maria Diaz de Haro 3, 48013 Bilbao, Spain*
[9]*Max Planck Institute for Chemical Physics of Solids, Dresden D-01187, Germany*

(Dated: October 25, 2021)

SUPPLEMENTARY NOTES

1. Introduction to supplementary notes

Supplementary notes (SN) are organized as follows. We extend our numerical examination of the bulk in SN 2-7, provide extra numerical investigations of the surface in SN 8-11 and specify details on the analytical models in SN 12-13.

2. Robustness of the topological gap

Here we show that fine tuning and perfect band folding are not strict requirements for achieving annihilation of Weyl points, endowing our system of a certain level of robustness and tolerance over reciprocal lattice vector mismatches. As shown in Supplementary Fig. 1, the annihilation of Weyl points, in the modulated system, occurs even under a bias field which is slightly different from finely tuned value allowing perfect band folding, i.e. when N_W is an integer. However, the band-gap is maximized at fine tuning and then gradually decreases when deviating from it. This indicated that the 3D CI phase can be achieved under a certain continuum interval of TRS breaking conditions centered around discrete values of the splitting.

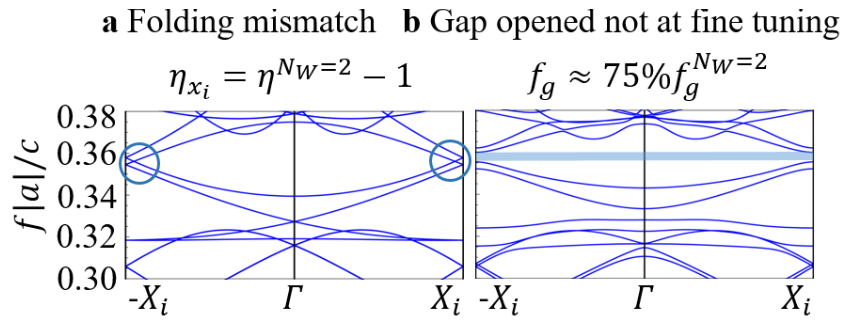

Figure 1: Weyl point annihilation occurring with tolerance over folding lattice vector and Weyl point location mismatch. **a** $(2, 2, 2)$ supercell with no supercell modulation: the gyro-electric parameter ($\eta^{N_W=2} = 15$) deviates from the finely tuned one ($\eta^{N_W=2} = 16$) required to merge Weyl points exactly at zone boundary. **b** After modulation, Weyl points annihilate, resulting in a topological band-gap f_g which is $\sim 75\%$ the maximum value $f_g^{N_W=2}$ obtained at fine tuning.

3. Minimizing the magnetic bias via multi-fold supercells

In the main text, we develop a novel strategy allowing us to generate the 3D photonic CI phase under more accessible magnetization conditions and minimal deformation of the bands. The method is based on the use of multi-fold supercells at small Weyl splitting: a 3D CI phase can be achieved under smaller splitting of the Weyl dipole as compared to the $N = N_W = 2$ system, by a proper choice of the modulation with $N = N_W > 2$. The detailed steps of this procedure are visualized in Supplementary Fig. 2, for an uniaxial supercell with $N = N_W = 3$. The resulting band-gap displays the same Chern number as the $N = N_W = 2$ system but it is achieved at a less than half the gyro-electric bias ($\eta_z^{N_W=3} = 7.8$, $\eta_z^{N_W=2} = 16$).

In order to decrease further the magnetic strength factor, in Supplementary Fig. 3 we provide some examples with larger $N = N_W$, in which we increased and optimized the modulation intensity in order to partially compensate for the band-gap decrease. The example case where $\eta/\epsilon = 1.6/16$ at $N = N_W = 7$, constitutes a significant reduction in the magnetic bias. However, we cannot indefinitely iterate this process down to zero magnetic field. Indeed, as $N = N_W$ grows, the band-gap suffers a reduction which is unavoidable: a decrease in the magnetic field necessarily leads to a compromise on the resulting gap.

To visualize this, we display a table of decreasing band-gaps for the uniaxial supercell where modulation intensity is chosen to maximize the band-gap at growing N, in Supplementary Table I. As it can be seen, the band-gap diminishes as $N = N_W$ grows. Clearly, in the limit of very large N, there is no splitting of Weyl points and thus no TRS broken gap can be opened, $\lim_{N\to\infty} f_g = 0$.

$N = N_W$	5	6	7
η	3.0	2.1	1.6
$f_g/f_m(\%)$	1.5	1.4	1.0
$f_g(f\|a\|/c)$	0.005	0.004	0.003

Table I: Reduction of the magnetic bias η and associated gap-to-midgap ratio $f_g/f_m(\%)$. Values are optimized via the modulation parameters, yet the band-gap reduction is unavoidable.

4. Higher Chern numbers on multi-fold supercells N>2

In the main text, we proved that arbitrarily large Chern numbers can be obtained by folding over multi-fold supercells with $N = 2n > 2$ and at $N_W = 2$. Supplementary Fig. 4 displays the band-structure associated to the WLs shown in Fig.**??** of the main text, for uniaxial supercells with N up to eight. As it can be noticed, the band-gap opens up at

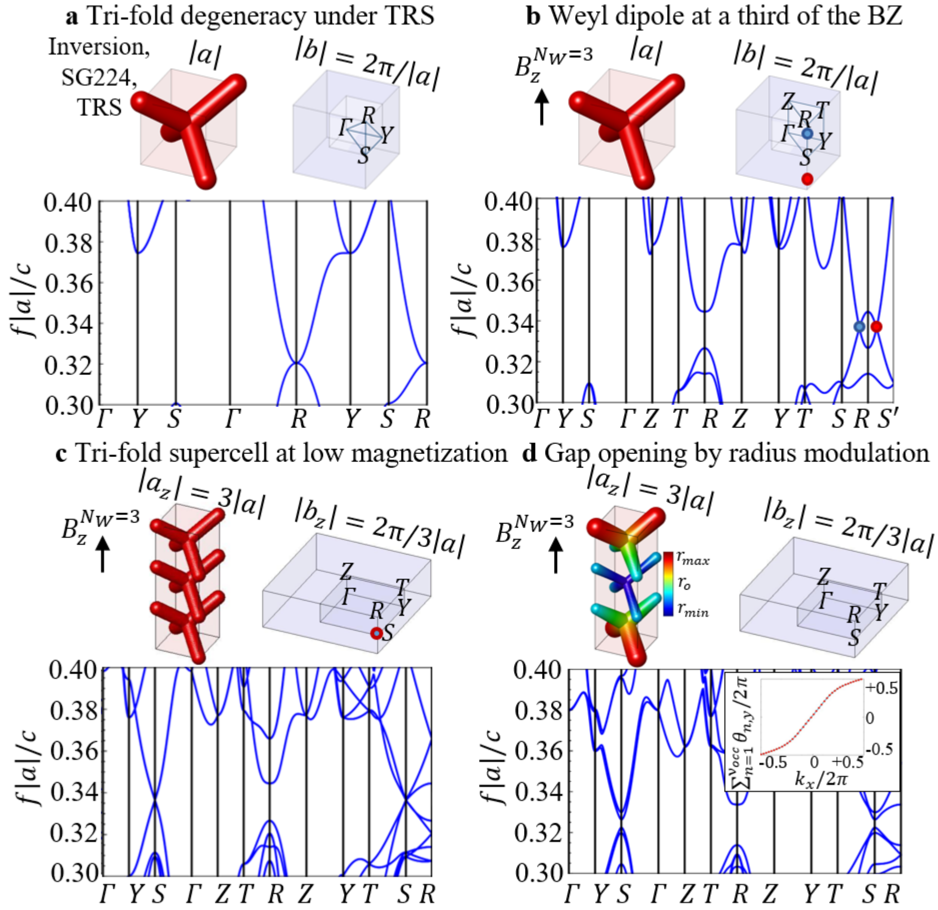

Figure 2: 3D CI in a reduced magnetization environment. Uniaxial supercell with $N = N_W = 3$, where $|a_z| = 3|a|$ is the lattice parameter the along z axis direction. **a** Photonic crystal in presence of TRS. **b** Limited TRS breaking implemented via a gyro-electric response to an applied $\mathbf{B} = B_z\hat{\mathbf{z}}$ field inducing a bias parameter $\eta_z^{N_W=3} = 7.8$: the bias field is adjusted in order to split the Weyl points of approximately a third the BZ, i.e. at $k_z^{\pm} = \pm\frac{2\pi}{3|a|}$, along the $\mathbf{SRS'}$ line where $\mathbf{S'} = \mathbf{S} - \mathbf{b}_z$. **c** Artificial folding of the bands on a $N = 3$ uniaxial supercell: Weyl points superimpose at $\mathbf{S}$ in the new BZ. **d** Coupling and annihilation of Weyl points through a $N = 3$ uniaxial supercell modulation with modulation parameter $r_m = r_0/20$, resulting in a topological direct gap at $\mathbf{S}$ with gap-to-midgap (f_g/f_m) ratio of 1.2%. The section Chern C_z number is constant everywhere in the BZ and displays unit value (inset), establishing the system to be in the same topological insulating phase as the $N_W = N = 2$ system, but with large reduction of the required magnetic bias.

$\mathbf{R} - \mathbf{Z} = \mathbf{S}$ if n is even and at $\mathbf{R}$ is n is odd. As the supercell modulation approaches a longer wavelength limit, the size of the gap gradually diminishes.

5. Cubic symmetry, Chern vector orientation and 3D CI/3D CI interfacing possibilities

The geometry of the crystal displays cubic symmetry even after the introduction of the supercell modulation, with three-fold rotational symmetry around the main diagonals of the cube. Therefore, as shown in Supplementary Fig. 5, it is possible to select each element of the first Chern class vector (C_x, C_y, C_z) by simply changing the orientation of the magnetization axis. Notice that, in order to avoid relabeling of the high symmetry points in the BZ at each different orientation of the magnetization axis, we stick to the labeling convention described in Supplementary Fig. 5(a). The existence of three weak indices in 3D allows for more interfacing possibilities as compared to the 2D case, where only the trivial/TI and the opposite (or different) Chern number combinations are meaningful (such as the anti-

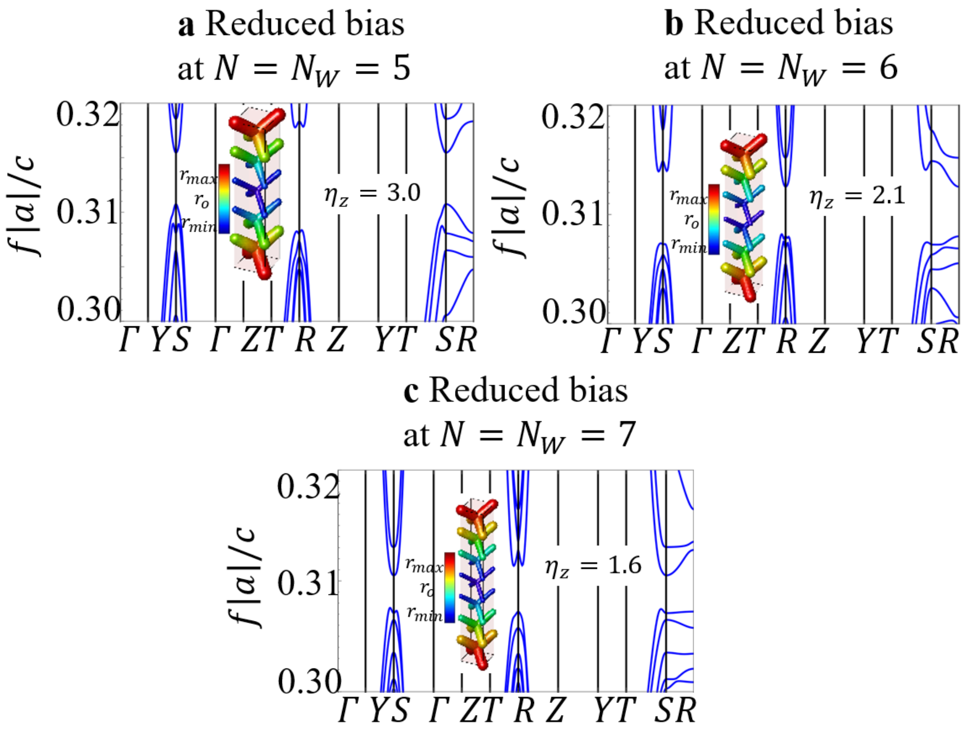

Figure 3: Reducing the magnetic strength factor by increasing the supercell size N and the modulation intensity. The band-gap undergoes a reduction in size.

ferromagnetic 2D C_zI/$-C_z$I interface). For example, considering that every planar cut parallel to the magnetization direction is capable of supporting anomalous surface states, it could be worth investigating what occurs at a 3D C_yI/ 3D C_zI planar interface. Similarly, owing to cubic symmetry of the 3D CI system, it would be possible to tune the relative angular phase in the supercell modulation in order to either break spatial inversion or not, which could lead to possible interesting surface states at the boundary of an obstructed atomic insulation (OAI) 3D CI and a 3D CI [1]. Another possibility allowed just in 3D, as proposed in Ref. [2], could be to arrange 3D CI around an inert core, with the 3D CI composing each panel having Chern vector (C_x, C_y, C_z) oriented to point inwards (e.g. fixing a 3D $+C_x$I on a left $\hat{\mathbf{x}}$ panel). Such a 3D interfacing arrangement, originally presented as a possible realization of a magneto-electrical (ME) coupler in the field of electronics, has no equivalent in photonics. Applications of this feature, originally analyzed in detail in [2], are left for further investigation. We leave the analysis of these interesting 3D interfacing possibilities for possible future investigations.

6. Details on the different modulation strategies

In this section we explain how to distinguish between different modulation strategies and better clarify the concept of supercell modulation. Uniaxial and cubic supercell modulated crystals are different in the following sense. In both cases the radius of the cylinders is locally varied, by locally changing the radius of the spheres in the covering approximation. However, the local change from the original r_0 radius to new local one $r(x, y, z)$ is performed according to the relation $\Delta r(x, y, z) = r_m \cos(2\pi x_i/N|a|)$, for the uniaxial case, while according to $\Delta r(x, y, z) = r(x, y, z) - r_0 = r_m[\cos(2\pi x/N|a|) + \cos(2\pi y/N|a|) + \cos(2\pi z/N|a|)]$, for the cubic case. More formally, one could introduce a vector of model parameters $\delta = (\delta_z, \delta_y, \delta_z)$ that differentiates the two situations as follows: $\Delta r(x, y, z) = r(x, y, z) - r_0 = r_m[\delta_x \cos(2\pi x/N|a|) + \delta_y \cos(2\pi y/N|a|) + \delta_z \cos(2\pi z/N|a|)]$ where $\delta = (1, 1, 1)$ for the cubic case where all components

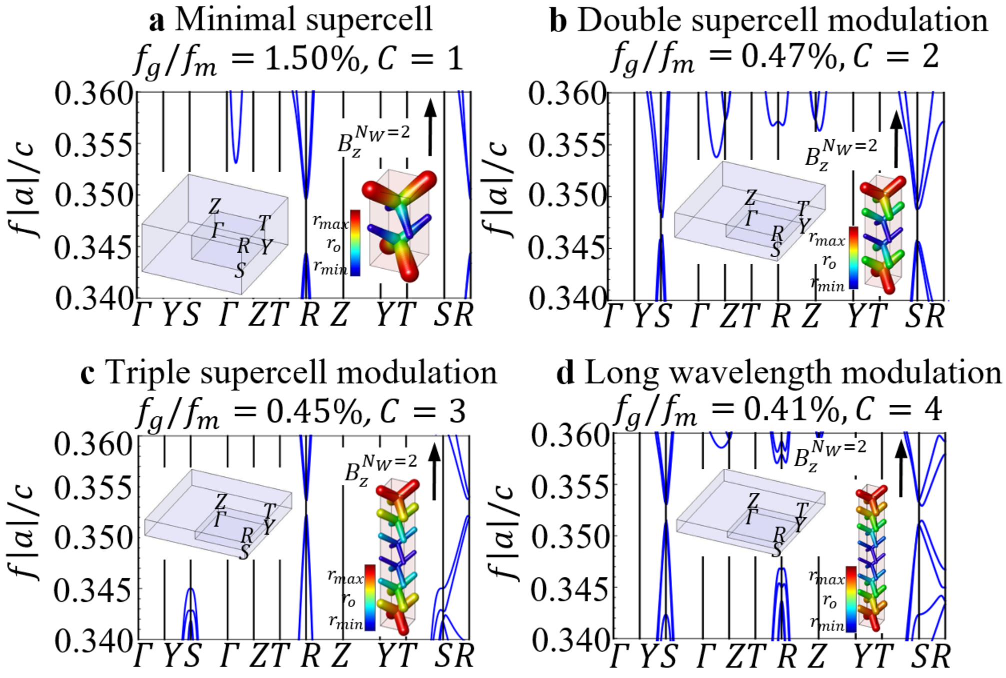

Figure 4: Band-structure for systems with increasing Chern number. Uniaxial $(1, 1, N)$ supercells with **a** $N = 2$, **b** $N = 4$, **c** $N = 6$ and **d** $N = 8$ and with modulation parameter $r_m = r_0/20$. The WLs are shown in Fig. **??** in the main text.

are modulated and $\delta = (0, 0, 1)$ for uniaxial modulation along $\hat{z}$. This means that, for an uniaxial supercell the modulation is performed along a single Cartesian axis while for a cubic one the structure is modulated along all the Cartesian components. To better differentiate the cases, here we report a plot 6 where the modulation parameter has been largely amplified $r_m \sim r_0/5$ and where we compare cubic and uniaxial supercells, on a (222) lattice so that one could better follow the 3D periodicity. Graphically, the supercell modulation is visualized by employing a scale of colors for the dielectric structure plots and a colorbar is associated to the local radius of the cylinders. Note that in the cases considered in the main text, the modulation applied is very little $r_m << r_0$, which may result in a very subtle graphical difference between between the original lattice and the modulated one.

7. Weyl dipole tilted from the Cartesian directions

The strategy we developed to obtain 3D Chern insulators still applies when the magnetic field deviates from the x, y, z symmetry lines, as long as the Weyl dipole is oriented along an integer linear combination of the lattice vectors. In such cases, a proper auxiliary supercell can be constructed that is commensurate with the Weyl dipole. For example, consider applying the magnetic field along (110). Since the starting point is a cubic lattice, we expect the Weyl points to split along the same direction, as in Supplementary Fig. 7; this relies on the fact that the system is a cubic lattice with O_h point group, and thus cyclic trifold symmetry in x, y, z. For a (110) Weyl dipole orientation, the proper auxiliary supercell should be have the following structure (N_x, N_y, N_z) with $N_x = N_y = N = N_W$ chosen to be commensurate with the Weyl dipole separation. For example, assume splitting the Weyl points at $N_W = 4$ with a field oriented along (110): by simple folding arguments, a band-gap can be opened via folding and modulating along (110) on a commensurate $N_x = N_y = N = 4$ supercell (either cubic with $N_z = N$ or anisotropic with $N_z \neq N$). This way one can achieve a Chern vector whose direction is not strictly a Cartesian direction provided being an integer linear combination of them. For the example discussed, we can expect the resulting system to have a non-zero Chern vector along (110). In general, the Chern vector should display the same orientation of the Weyl dipole, as long as

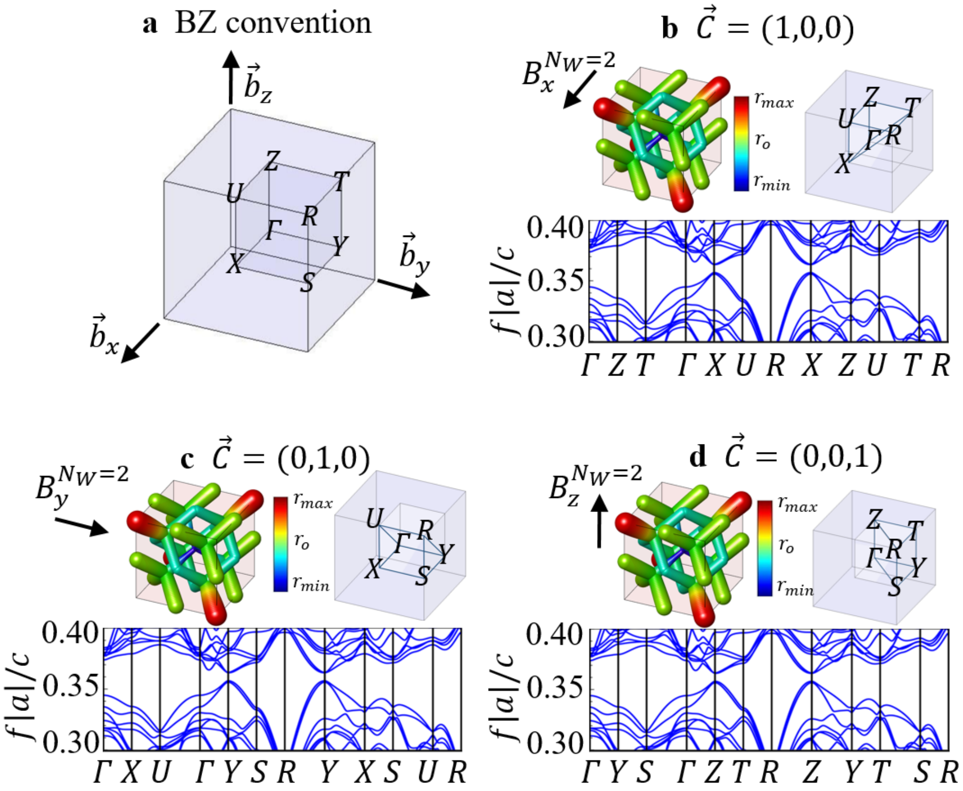

Figure 5: Orientability of the Chern vector $\vec{C} = (C_x, C_y, C_z) \equiv C_x\hat{\mathbf{x}} + C_y\hat{\mathbf{y}} + C_z\hat{\mathbf{z}}$ with the external magnetic field. **a** Labeling convention of the BZ adopted in the main text, to capture all symmetry and orientations of the designed unit cells: each point $\mathbf{X}_i = \frac{\mathbf{b}_i}{2}$ is indicated according to the corresponding primitive reciprocal lattice vector $\mathbf{b}_i$, i.e. in a orthorhombic notation. **b,c,d** $(2, 2, 2)$ supercells: owing to cubic symmetry of the supercell modulation, a 3D Chern insulating phase can be obtained for any choice of the magnetization along the three principal axes; shown the corresponding tetragonal IBZs for $\hat{\mathbf{x}}, \hat{\mathbf{y}}, \hat{\mathbf{z}}$ respectively.

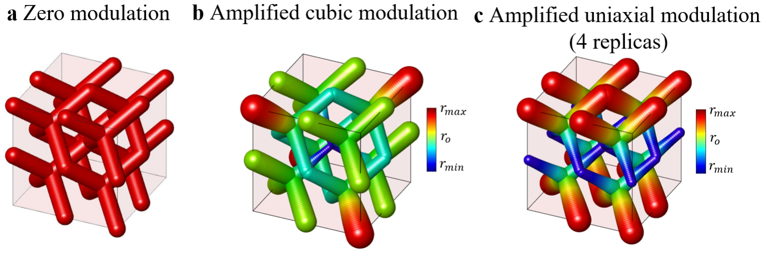

Figure 6: Visualizing the supercell modulation when the model parameters are largely amplified $r_m \sim r_0/5$. Cubic (**b**) and uniaxial case (**c**). For the uniaxial case, we replicate laterally 4 unit cells, in order to better compare the 3D periodic structure with the cubic one.

one chooses a commensurate supercell which annihilates them.

8. 3D CI/Trivial insulator interface

In order to observe topological edge states at the interface formed by a trivial insulator and the 3D CI, we search for a photonic crystal with a band-gap completely overlapping the topological system one. Indeed, finding a proper

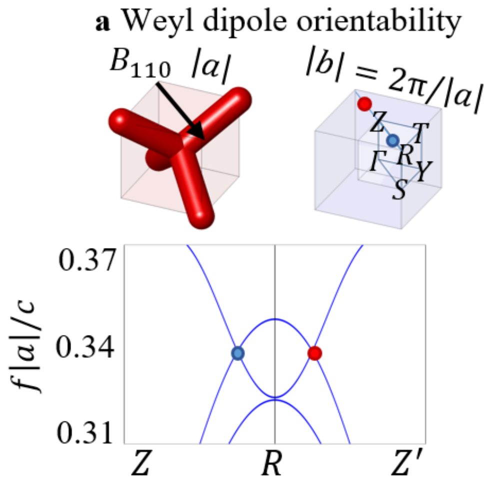

Figure 7: Orienting the Weyl dipole along an an integer linear combination of the Cartesian components. Splitting occurs along the $\mathbf{ZRZ'}$ line where $\mathbf{Z'} = \mathbf{Z} - \mathbf{b}_y - \mathbf{b}_x$

insulating interface is a necessary requirement in order to prevent propagation of edge modes in free space due to modes living in the light cone and to avoid formation of dangling defects states due to lattice mismatches: this is usually a quite difficult task in 3D, due to limited available band-gap geometries as compared 2D. In this work, this is achieved by means of a triply periodic minimal surface (the plumber's Schwarz P [3]) having the spatial symmetries of SG No. 221 and displaying a large trivial gap. This photonic crystal consists of an isotropic TRS dielectric material

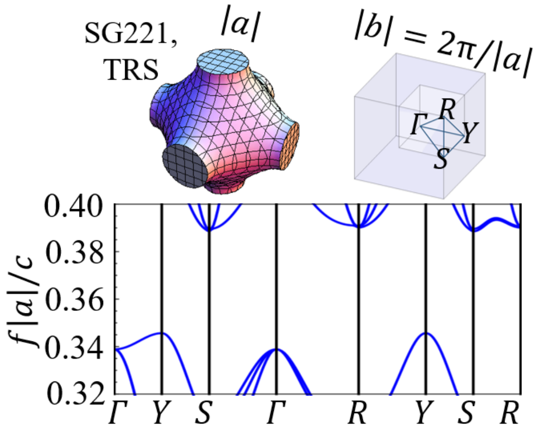

Figure 8: Insulating trivial interface consisting of dielectric material $\varepsilon_{triv} = 16$ embedded in a plumber Schwarz P minimal surface (SG 221) with $c = 0.5$. The trivial gap contains completely with the gap of the topological system.

$\varepsilon{=}\varepsilon_{triv}\mathbb{1}_3$ embedded in the region of space defined as $g(x, y, z) < h$ where $h \in \mathbb{R}$ and $g(x, y, z)$ is the triply periodic minimal surface: $g(x, y, z) = \cos(2\pi x/|a|) + \cos(2\pi y/|a|) + \cos(2\pi y/|a|)$. As shown in Supplementary Fig. 8, the trivial photonic gap contains the topological band-gap of 3D CI completely.

9. Absence of Chiral Magnetic Effect (CME) or absence of any net energy transport along the magnetization direction

To investigate the possibility of SS energy propagation along the magnetization axis, we consider the relation existing between the Poynting vector $\mathbf{S}$ and the group velocity $\mathbf{v}_g$ in lossless, dispersive media $\mathbf{S} = W\mathbf{v}_g$ $(W > 0)$ [4]. By integrating the component of the group velocity parallel to the magnetization axis $\mathbf{v}_g \cdot \frac{\mathbf{B}}{|\mathbf{B}|}$ over the entire surface plot of Fig. **??**, we concluded that no net energy transport along the magnetization direction can be achieved. This is expected since in equilibrium, no chiral magnetic effect (CME) can be observed [5].

10. Optical chirality

By evaluating the polarization state of each edge channel, we observed a well defined sign of the average optical chirality $\bar{c} > 0$ though the entire SS. The average optical chirality $\bar{c}$ is defined as a spatial average of the density of optical chirality c [6], over the region occupied by the edge modes as follows:

$$\bar{c} = \frac{\int_{edge} d^3\mathbf{r} P(\mathbf{r}) c(\mathbf{r})}{\int_{edge} d^3\mathbf{r} P(\mathbf{r})} \tag{1}$$

where $c(\mathbf{r}) = Z_0 \cdot \mathrm{Im}(\mathbf{E}^* \cdot \mathbf{H})$ is the density of optical chirality and Z_0 the vacuum impedance; The projectors $P(\mathbf{r})$, defined as $P(\mathbf{r}) = 1$ if $\varepsilon(\mathbf{r}) = \mu(\mathbf{r}) = \mathbb{1}_3$ and 0 elsewhere, are introduced in order to only take account of the regions where electromagnetic duality is preserved and the optical chirality is ensured to be physical conserved quantity [6]. This quantity was computed separately using the Bloch wavefunction of each modes with momentum $\mathbf{k}$ and band m in the SS and for opposite values of the applied $\mathbf{B}$ magnetic field. In conclusion, the optical chirality was found to be even under TRS: indeed, it did not change under reversing either the external $\mathbf{B}$ field or the group velocity of the edge channel $\mathbf{v}_g$, consistent with the bosonic nature of the light [6]. Moreover we obtained consistent values for $\bar{c}$ among all the edge channels at midgap. A definite electromagnetic chiral character of the surface states could find applications in the emergent field of chiral light matter interactions [7–11].

11. Evolution of WS Fermi arcs into 3D CI Fermi loops

In this section we show that the Fermi loops of the 3D Chern phase naturally evolve from the Fermi arcs [12] of the starting photonic Weyl semimetal setup as the Weyl points undergo annihilation. In particular, we investigate the fate of the Fermi arcs and describe their evolution during the supercell folding and modulating process.

Our results can be summarized by Supplementary Fig. 9, for an uniaxial system with $N = N_W = 2$. Panel A shows the Fermi arcs of the Weyl semimetallic phase. Opposite charged Weyl points are located at $(k_y^{\pm}, k_z^{\pm}) = (\pm\frac{\pi}{2}, \pi)$, i.e. midway along the **SR** line. After supercell folding, the Weyl points are superimposed and their Fermi arcs display a folding as well, as in panel B. Finally, by turning on the supercell modulation, a gap is opened in the BZ and the Fermi arcs sharply evolve into disjoint Fermi loops of the 3D CI system, as in panel C. Therefore, the Fermi arcs of the Weyl semimetal are transformed into the Fermi loops of the 3D CI system as result of the supercell folding and modulation.

In addition, in order to show a smoother evolution of Fermi arcs into loops, we also compute them for a system where we have reversed the order of TRS symmetry breaking and supercell modulation. Thus, in this new set of calculations, we first spatially modulate our structures and then we apply the magnetic field adiabatically increasing its value until the Weyl points annihilate. In this case, as shown in Supplementary Fig. 10, Fermi points appear in the modulated system as small TRS breaking is introduced (panel A), they gradually move far apart as the magnetic bias is increased with their Fermi arcs getting longer (panel B), up to final annihilation in panel C.

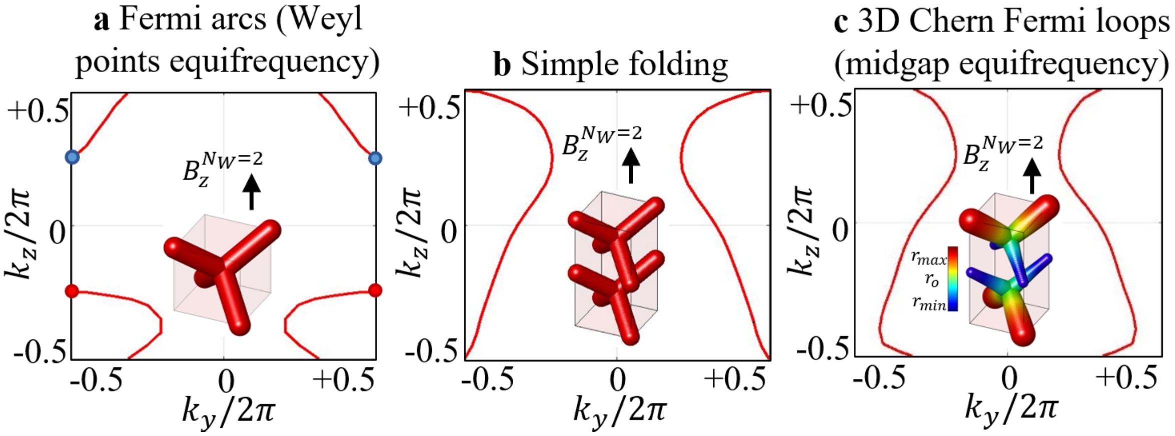

Figure 9: Evolution of the Fermi arcs (**a**) after Brillouin zone folding (**b**) and supercell modulation (**c**).

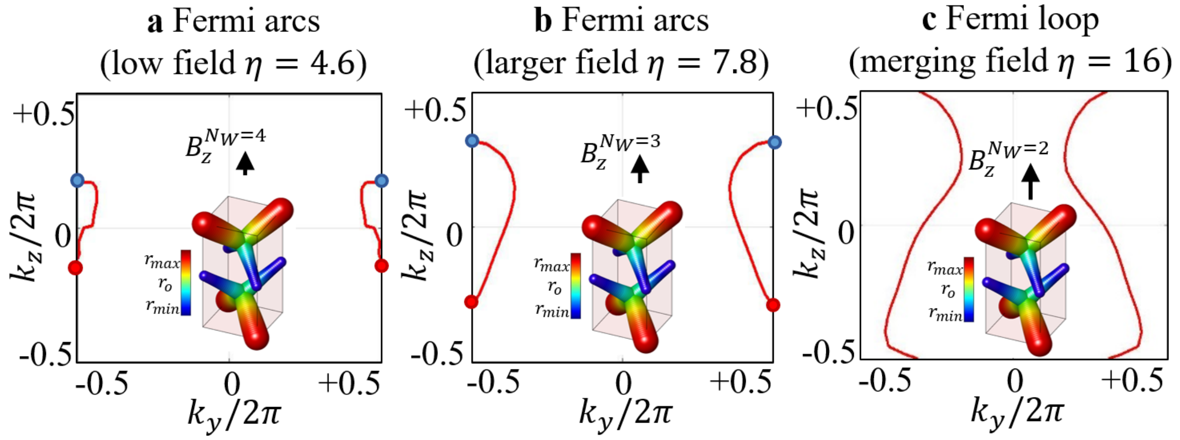

Figure 10: Evolution of the Fermi arcs reversing the order of TRS breaking and modulation. The starting setup is a supercell modulated system with small magnetic field (**a**). As they field is increased the Weyl point separations growns (**b**). Finally Weyl points annihilate and Fermi arcs evolve into Fermi loops (**c**).

12. Analytical model for coupled Weyl points: Case $N = N_W = 2$

In Eq. **??**, we give the expression for the modulation transforming as the X_1 irrep of the little group at $\mathbf{X}_3 = (0, 0, 1/2)$. Here, we present the full expression including modulations that where not used in the model because they would not open a gap (as shown in Supplementary Fig. 11 for a generic choice of parameters) and that are TR-even.

For brevity, we provide the 3×3 block, analogous to X_1 in Eq.**??**:

$$X = \begin{pmatrix} C_1(p_1 - iq_1) & ip_3 + q_4 & -iq_3 + p_4 \\ -ip_3 - q_4 & C_2p_1 - iC_3q_1 & ip_2 + q_2 \\ -iq_3 + p_4 & -ip_2 - q_2 & -iC_2q_1 + C_3p_1 \end{pmatrix}, \tag{2}$$

where (p_m, q_m) are real and belong to the 2D irreps X_m and $C_{1,2,3}$ are real coupling constants for the X_1 modulation.

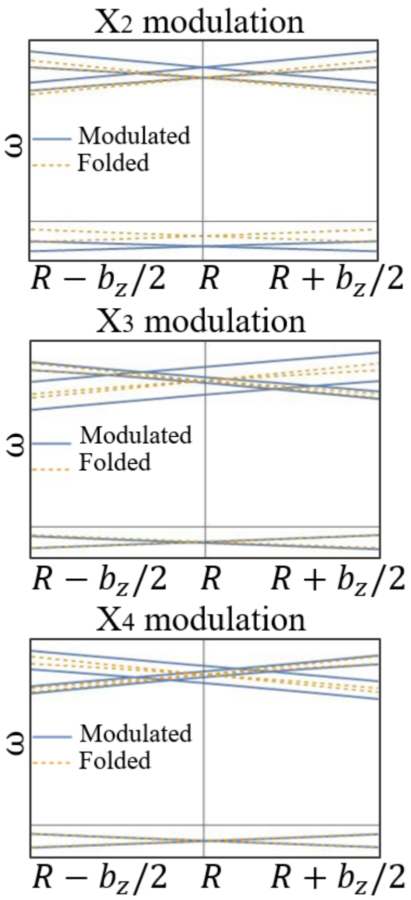

Figure 11: Examples of perturbations that do not open a gap. **a,b,c** modulations belonging to irreps X_2, X_3 and X_4 respectively.

13. Analytical model for coupled Weyl points: Case $N > 2$

In these cases, the modulation belongs to the high-symmetry line $\mathbf{\Delta} = (0, 0, v)$ so the little group of its wave vector is smaller than in the $N = 2$ case. The expression for the diagonal blocks in Eq. **??** is identical, since the Weyl points still lie on the $\mathbf{T}$ line, but the reduced symmetry of the modulation modifies the off-diagonal blocks. The most general expression for TR-even modulations in this case is the following:

$$\Delta = \begin{pmatrix} A_1 r_3 & -r_5 & i s_5 \\ r_5 & r_1 + A_2 r_3 & i r_4 \\ i s_5 & -i r_4 & -r_1 + A_2 r_3 \end{pmatrix}, \tag{3}$$

where r_l and s_l belong to the Δ_l $(l = 1, 3, 4, 5)$ irreps and all of them are complex. Note that (r_5, s_5) is the basis for the 2D irrep Δ_5 and $A_{1,2}$ are real coupling constants for the Δ_3 modulation.

SUPPLEMENTARY REFERENCES

* chiara.devescovi@dipc.org
† maiagvergniory@dipc.org
‡ aitzolgarcia@dipc.org

[1] Benjamin J Wieder, Kuan-Sen Lin, and Barry Bradlyn. Axionic band topology in inversion-symmetric weyl-charge-density waves. *Physical Review Research*, 2(4):042010, 2020.
[2] David Vanderbilt. *Berry Phases in Electronic Structure Theory: Electric Polarization, Orbital Magnetization and Topological Insulators*. Cambridge University Press, 2018.
[3] L Martin-Moreno, FJ Garcia-Vidal, and AM Somoza. Self-assembled triply periodic minimal surfaces as molds for photonic band gap materials. *Physical review letters*, 83(1):73, 1999.
[4] Said M Mikki and Ahmed A Kishk. Electromagnetic wave propagation in nonlocal media: Negative group velocity and beyond. *Progress In Electromagnetics Research*, 14:149–174, 2009.
[5] P Baireuther, JA Hutasoit, J Tworzydło, and CWJ Beenakker. Scattering theory of the chiral magnetic effect in a weyl semimetal: interplay of bulk weyl cones and surface fermi arcs. *New Journal of Physics*, 18(4):045009, 2016.
[6] Lisa V Poulikakos, Jennifer A Dionne, and Aitzol García-Etxarri. Optical helicity and optical chirality in free space and in the presence of matter. *Symmetry*, 11(9):1113, 2019.
[7] Aitzol García-Etxarri and Jennifer A Dionne. Surface-enhanced circular dichroism spectroscopy mediated by nonchiral nanoantennas. *Physical Review B*, 87(23):235409, 2013.
[8] Chi-Sing Ho, Aitzol Garcia-Etxarri, Yang Zhao, and Jennifer Dionne. Enhancing enantioselective absorption using dielectric nanospheres. *ACS Photonics*, 4(2):197–203, 2017.
[9] Michelle L Solomon, Jack Hu, Mark Lawrence, Aitzol García-Etxarri, and Jennifer A Dionne. Enantiospecific optical enhancement of chiral sensing and separation with dielectric metasurfaces. *ACS Photonics*, 6(1):43–49, 2018.
[10] Jon Lasa-Alonso, Diego Romero Abujetas, Álvaro Nodar, Jennifer A Dionne, Juan José Sáenz, Gabriel Molina-Terriza, Javier Aizpurua, and Aitzol García-Etxarri. Surface-enhanced circular dichroism spectroscopy on periodic dual nanostructures. *ACS Photonics*, 7(11):2978–2986, 2020.
[11] Aitzol García-Etxarri, Jesus M Ugalde, Juan Jose Sáenz, and Vladimiro Mujica. Field-mediated chirality information transfer in molecule–nanoparticle hybrids. *The Journal of Physical Chemistry C*, 124(2):1560–1565, 2019.
[12] Shuang Jia, Su-Yang Xu, and M Zahid Hasan. Weyl semimetals, fermi arcs and chiral anomalies. *Nature materials*, 15(11):1140–1144, 2016.
